# Hospital Penny Fund

**Published:** 1904-11-19

**Authors:** 


### HOSPITAL PENNY FUND.

### REORGANISATION MEETING ADJOURNED.

A meeting was convened on Tuesday last at the Blenheim Rooms, Hôtel Cecil, by the promoters of the Hospital Penny Fund, at which some 20 gentlemen were present, among them being the following:—

Sir Anthony Crompton Thornhill (in the chair), Sir Edward Noel Walker, Messrs. George Herring, P. Michelli (Seamen's Hospital), G. A. Arnaudin (St. John's Hospital for Skin Diseases), Alfred Craske (North-West London Hospital), James McKay (Great Ormond Street Hospital for Children), Irwin H. Beattie (St. Peter's Hospital), A. J. Borer (Tilbury Hospital), G. Owen Ryan, W. Rotherham, F. W. E. Clark, A. W. Scrivener (West Ham Hospital), P. Salmon, G. H. Page, C. E. Warren, F. Stokes, and H. M. Goldman (secretary).

The Secretary read a letter which had been sent to the authorities of all the London hospitals, inviting their attendance for the discussion of a scheme for the reorganisation of the Fund.

The Chairman said that notices had been sent to all the London hospitals, and he had hoped to see their representatives present at that meeting. There had been a good deal of controversy and a good deal of misapprehension with regard to the scheme, although all the details had been supplied in circulars and letters that had been sent out. It seemed to him that the great point to be considered by the hospital authorities was that by the introduction of this Fund large sums ought to be secured for the hospitals. Unfortunately it had not been well received. They had been able to approach a gentleman well known in philanthropic work — Mr. George Herring — and it was due to his suggestion that the reorganisation scheme had been brought forward. The Hospital Penny Fund would not encroach on the field of any other organisation. It was apparent that the 25 per cent. proposed to be taken for expenses was barely sufficient for the purpose. They were sorry there was a lack of response on the part of people well known to the hospital world; that was why they had called that meeting and invited hospital representatives to it. They wanted them to elect trustees for the Fund, and also a committee of hospital representatives to advise on all matters connected with it. He regretted that Mr. Drane, the managing director of *Public Opinion*, had contracted an attack of laryngitis, or he would have been present to answer any questions they might wish to put to him.

Sir Noel Walker said that at the meeting held elsewhere in the month of May he was unexpectedly called on to preside, and he came before them that day in somewhat similar circumstances, but any want of information he might show was, he felt, made up for by his interest in the matter. He had been a member of the committee, but that had been dissolved, and so he had now no *locus standi.* He had, however, expressed his willingness to come and give any explanation he could. He wished to stand by his late colleagues; he wished to share any odium they might have incurred and the defence which they were almost called upon to make. He thought the scheme was a good and honest one which had been undeservedly condemned. He wanted to know what had been their offence. Two points had been put forward. One was that the committee was not sufficiently influential; the other was that someone was supposed to be exploiting the hospitals for private purposes. On the first point, as to the unsuitability of the committee, he entirely and fully agreed. He himself joined it at first regarding it much as he would a Church bazaar, and considering it equally objectionable; but when he looked into it he found that in the opinion of many people it was a scheme that might do good work in securing pennies and availing itself of the prevailing mania for collections and competitions. It still seemed to him that this was harmless enough, and if there were any greater objections to it than to Church bazaars he should like to hear what they were. As to the second point, that someone was getting something out of it, they all knew as well as he did that they could not have any scheme without paying for it. He had joined without knowing where the money was coming from. He felt sure that so far the financial promoters had come off very badly. None of those before him, he was sure, worked for nothing, and he himself had worked for a salary all his life. He had had the advantage of looking behind the scenes, and had satisfied himself that 25 per cent. was not an unreasonable sum for expenses and that no one would be able to do it for less. Let them look at it as a mere commercial transaction; if they went, say, to a Universal Provider, a hospital would be very glad if he would raise £1,000,000 and take £250,000 for doing it, or if he raised £100,000 and took £25,000. All he and his colleagues as a committee had to do was to see that the 75 per cent. was properly applied; strictly speaking, they had nothing to do with the other 25 per cent. However, they made it a condition that they should look into the 25 per cent. to see that they were not connected with anything discreditable. They had not found that there was the slightest objection to any inquiry, and he should like to repeat the opinion he had expressed that this was a perfectly fair and honest and quite unobjectionable scheme.

Mr. George Herring, who was received with applause, said that in his idea the scheme was a good scheme in itself and tapped a source not yet touched. But, if the hospitals said they were against the scheme and said they wouldn't take the money, he could not see how it was to go on. He should have thought they would take it and be thankful. For instance, if anyone said he would start a bazaar in aid of a hospital he should say Certainly, if it were run on a good principle. What was apparent about this scheme was that here was an offer of about £5,000, with all the expense and risk in connection with it, for the purpose of helping the hospitals. There might be something in the background of which he did not know, and he had come there to hear what the opponents of the scheme had to say about it. (Applause.)

The Chairman here invited any representative of a hospital present to give them the benefit of his views. After a pause, Mr. Michelli (of the Seamen's Hospital, Greenwich) said the views of representatives of the hospitals were invited, but his colleagues were not there. He saw representatives of some of the junior hospitals present, but the 12 great hospitals were missing and so were the eight others who had representatives on the Central Hospital Board. He recalled that they had the same state of things at the meeting at Henrietta Street. He did not go there prepared to oppose any scheme for raising money if it were a good one. He was a paid beggar himself and ready to get money for the Seamen's Hospital by any proper means. Before he went to that meeting, in consultation with his colleagues he arranged to ask two questions: Who is there connected with this scheme well known in the hospital world whom we may follow and have confidence in? and Who is backing and financing the scheme? To both questions he got unsatisfactory replies. To the first he was refused an answer, and to the second one had never been forthcoming. Two gentlemen had called upon him at his office, and had led him to understand that they had obtained the support of some of the hospitals, but on inquiry over the telephone he found that was false, and that they had not the least ground for the assertion. He expected to find on the notices sent out the names of gentlemen recognised as authorities, but the only name that appeared was that of Mr. Goldman, a name unknown to him and unknown in the hospital world. He asked now, as he had asked before, who was financing this scheme. Already, apparently some £600 had been spent. The promoters must be open and candid. He saw no objection to the scheme *per se*, but when he asked Mr. Holland, Sir Henry Burdett, Mr. Danvers Power, Sir Savile Crossley, men whom they looked up to and trusted for guidance, they said Have nothing to do with it. Certainly the Hospital Penny Fund had one good card in Mr. Herring, than whom nobody in the hospital world commanded greater respect—(hear, hear)—and his connection with it gave them pause. Well, the hospital secretaries of London put their heads together; the matter was discussed at the Central Hospital Board for London, on which were represented the 20 great hospitals, and that Board unanimously resolved that they would not associate themselves with this scheme. They wanted to know who was responsible for it. Sir Noel Walker had compared the scheme to a company asking for money. Yes, but the prospectus would have names on it, and unless it did the promoters would not get their money. He wished to say nothing about the scheme itself, but they must know more about the people connected with it before they could give it their confidence. Others there he understood were prepared to criticise the scheme on its merits, being doubtful as to whether it could pay its way, but with that aspect he was not now prepared to deal.

Mr. Herring said that with every word of what Mr.

Michelli had said he agreed. He suggested that a number of hospital delegates should meet and nominate trustees to receive the money, should put an accountant to check the accounts, and so put the matter on a proper basis. He quite agreed that the scheme had come before the public in a stupid manner, but if the scheme itself were good it might now be reclaimed by bringing in the hospital authorities to take charge of the money and accounts and choosing trustees and a committee from among them.

Mr. Herring said he distinctly understood that the promoters were to find the prize money and received the Chairman's assurance that they had already found it.

Mr. Michelli: We ask who are finding it?

The Chairman: If the money is provided is there any need to put forward names? The trustees will have the money for the prizes handed over to them each month.

Mr. McKay said they would not find the hospitals joining the scheme unless they were given the names of the people responsible.

At this point conversation and question and answer became general all over the room with nothing definite emerging which could be followed; after which

Sir Noel Walker said it seemed a pity that a scheme, about which there appeared to be a general agreement that it was a good one, should fall through. The money was there, but he was certain that was not the only objection. He was perfectly disinterested in the matter, as he had no intention of being further connected with it.

It was ultimately suggested by the representative of the West Ham Hospital that a full report of the proceedings that day should be sent to the authorities of the London hospitals and to the Central Hospital Board, so that their views might be obtained, and on the proposal of Mr. Craske the meeting was adjourned until that had been done, it being understood that the promoters were willing to disclose the name of the donor of the bonus money to two or three representative men, and to put the money in the hands of trustees to be chosen by the hospitals.

∴ The meeting ignored the real points at issue. They are that the hospitals are already reaping all the benefits that stamps can confer upon them through the League of Mercy, without deducting 25 per cent., or indeed one farthing, from the money paid by the public for the King's Fund stamps. That the proposals have been proved by the experience of the King's Fund to be impracticable, as the public will not purchase labels miscalled stamps. That any hospital desiring to try and raise funds in this way can obtain the stamps free through the League of Mercy, and so secure the whole proceeds for their own benefit. That the hospitals cannot be exploited for the benefit of any promoter, who, by offering prizes, hopes to raise a sufficient sum to recoup his outlay and leave a profit for himself, as indicated by Sir Noel Walker. The meeting was a fiasco, and the Hospital Penny Fund should be allowed to die at once.—Ed. The Hospital.

